# The Use of TAT Peptide-Functionalized Graphene as a Highly Nuclear-Targeting Carrier System for Suppression of Choroidal Melanoma

**DOI:** 10.3390/ijms20184454

**Published:** 2019-09-10

**Authors:** Suyan Shan, Shujuan Jia, Tom Lawson, Lu Yan, Mimi Lin, Yong Liu

**Affiliations:** 1 Laboratory of Nanoscale Biosensing and Bioimaging, School of Ophthalmology and Optometry, School of Biomedical Engineering, Wenzhou Medical University, 270 Xueyuanxi Road, Wenzhou 325027, China; 2 ARC Center of Excellence for Nanoscale Bio Photonics, Macquarie University, Sydney, NSW 2109, Australia

**Keywords:** trans-activating transcriptional activator (TAT) peptide, graphene, mitomycin C (MMC), nuclear targeting drug delivery, choroidal melanoma

## Abstract

Tumorous metastasis is a difficult challenge to resolve for researchers and for clinicians. Targeted delivery of antitumor drugs towards tumor cells’ nuclei can be a practical approach to resolving this issue. This work describes an efficient nuclear-targeting delivery system prepared from trans-activating transcriptional activator (TAT) peptide-functionalized graphene nanocarriers. The TAT peptide, originally observed in a human immunodeficiency virus 1 (HIV-1), was incorporated with graphene via an edge-functionalized ball-milling method developed by the author’s research group. High tumor-targeting capability of the resulting nanocarrier was realized by the strong affinity between TAT and the nuclei of cancer cells, along with the enhanced permeability and retention (EPR) effect of two-dimensional graphene nanosheets. Subsequently, a common antitumor drug, mitomycin C (MMC), was covalently linked to the TAT-functionalized graphene (TG) to form a nuclear-targeted nanodrug MMC-TG. The presence of nanomaterials inside the nuclei of ocular choroidal melanoma (OCM-1) cells was shown using transmission electron microscopy (TEM) and confocal laser scanning microscopy. In vitro results from a Transwell co-culture system showed that most of the MMC-TG nanodrugs were delivered in a targeted manner to the tumorous OCM-1 cells, while a very small amount of MMC-TG was delivered in a non-targeted manner to normal human retinal pigment epithelial (ARPE-19) cells. TEM results further confirmed that apoptosis of OCM-1 cells was started from the lysis of nuclear substances, followed by the disappearance of nuclear membrane and cytoplasm. This suggests that the as-synthesized MMC-TG is a promising nuclear-target nanodrugfor resolution of tumorous metastasis issues at the headstream.

## 1. Introduction

New drug delivery systems are required thatare highly active in the body and have a high specific targeting capability. This goal is considered an ultimate outcome for chemotherapy. For example, the biggest challenge for the clinic utilization of ophthalmic drugs is their low bioavailability as the blood retina and the cornea act as barriers [1]. Novel nanomaterials may penetrate these physiological barriers and may offer opportunities for the preparation of novel ocular drug delivery systems that can effectively target specific sites of importance. To this end, various biocompatible nanomaterials have been developed to serve as nanosized drug carriers for chemotherapy. These include carriers made of metals, metal oxides, semiconductors, and polymers [2,3,4,5]. Some of these have realized the targeted delivery of nanodrugs to tumors’ microenvironments and their cellar membranes, but their efficiency is still desired. Many factors have caused this, such as the content of protein and molecules on the surface of membranes and changes on the cellar microenvironment. Less than good results have led to the low targeted suppression of tumors during clinic trials of these new nanodrug carriers. Thus, it continues to be important to develop new nuclear-targeting systems that can deliver antitumor drugs to tumor cells. A nuclear-targeting drug delivery system is seen as one efficient approach to improving the efficiency of the targeted suppression of tumors during these clinical trials [6]. Furthermore, this approach offers additional advantages as they provide metastatic inhibition of tumors. It is commonly known that cell nuclei are the source of gene inheritance and transcription [7]. 

Choroidal melanoma (CM) is a primary ocular malignant tumor observed frequently amongst adults. Although numerous techniques, including resection, enucleation, radiotherapy, and laser therapy, can be used to treat CM, significant side effects from these treatments are inevitable [8,9]. For example, surgery such as the resection and enucleation of an eyeball, can cause severe pain for patients. Radiotherapy increases the likelihood of CM relapse. When laser therapy phototherapy is used, its impact is limited to the location of tumors. Treated patients still suffer from low long-term survival rates and a high risk of tumor metastasis [10]. The biggest problem in CM therapy is its ease of metastasis. The five-year survival rate of CM is less than 50% when tumor cells do metastasize [11]. Systemic chemotherapy was reported elsewhere as an effective tool to suppress the deterioration of CM [12]. However, the easy metastatic characteristic of CM is still a high risk in a majority of patients with the disease. The development of nanomaterials or small-molecule-based nuclear-targeting techniques may provide a solution to the poor outcomes that most patients experience.

In this work, a novel nanoscale drug delivery system was formulated and had a high nuclear-targeting capacity. This property was observed after combining the advantages of the unique size of graphene materials with the properties of trans-activating transcriptional activator (TAT). Graphene-based nanomaterials (particularly those that are less than 200 nm in diameter) can pass through the leaky tumor vessels and can accumulate in the tumor and in its microenvironment. This is due to the enhanced permeability and retention (EPR) effect of nanomaterials [13,14]. In this work, we report the formulation of TAT with graphene to form a composite that has an improved targeting capability for nuclei and that shows an improved biocompatibility. TAT is a kind of cationic cell penetrating peptide (CPP) that is composed of short amino acid sequences. It is able to penetrate the biological membrane and to translocate to the nucleus [15]. Furthermore, TAT is known for its non-immunogenicity and low cytotoxicity properties [16].

In the present work, nanosheets from TAT-functionalized graphene (TG) were prepared via an efficient edge-functionalized ball-milling (EFBM) method as reported by the author’s research group previously [17,18]. TAT aids in triggering the nuclear targeting of tumors during the administration of TG. The hydrophobicity and biocompatibility of TG was also improved by the use of TAT. The as-prepared TG was then tested as a nanocarrier for the immobilization of the antitumor drug mitomycin C (MMC). MMC demonstrated elsewhere a capability to inhibit DNA synthesis of tumor cells [19]. The resulting nanodrug from MMC-loaded TG (MMC-TG) demonstrated elsewhere a good tumorous nuclear-targeting capability and a strong suppressing capability on tumor cells. The successful penetration of MMC-TG through the tumorous cell membrane and the nuclear membrane was confirmed. The apoptosis of tumor cells was found to start from lysis of the nuclei, and was followed by the dissolution of karyotheca and cytoplasm. These results suggest the usefulness of the as-synthesized TG-based system as a highly efficient nuclear-targeting antitumor drug-carrier and as a possible treatment to reduce the metastatic properties of malignant tumors.

## 2. Results

**Figure 1:**
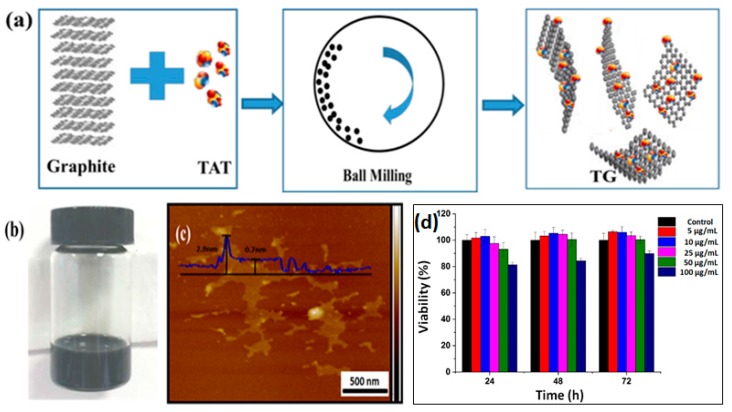
(**a**) A schematic synthesis of trans-activating transcriptional activator (TAT) peptide-functionalized graphene (TG) via the ball-milling method, (**b**) digital image of the TG well dispersed in deionized water, (**c**) atomic force microscopy (AFM) micrograph of the as-prepared TG, and (**d**) the cell viability of ARPE-19 cells incubated with TG for various lengths of time.

Figure 1a exhibits a schematic synthesis of TG via the EFBM method. At the initial step of ball-milling, TAT functionalized the edge of graphite sheets via a solid mechanochemical reaction that was reported elsewhere [20,21]. The amount of TAT linked to the layered graphite edge increased gradually with milling time. The TAT content facilitated layered space expansion and a decrease in bond energy between each layer. This, along with the milling shear forces, caused the exfoliation of graphene-based nanosheets. It is well-known that graphene is a hydrophobic material. When TAT was introduced into graphene, however, the resulting TG was observed to have a high hydrophilicity. As shown in Figure 1b, the as-prepared TG was dispersed in deionized (DI) water. This dispersion showed no visible aggregation over 7 days. The microstructure of TG was observed using atomic force microscopy (AFM) and is shown in Figure 1c. The average thickness of the as-prepared graphene nanosheets was approx. 0.7 nm, indicating the successful preparation of single-layered nanosheets. At the edge of the sheets, some bright nanomaterials with a thickness of 2.9 nm on the nanosheets were also visible. This indicated the presence of TAT attached to the resulting TG. AFM results confirmed the successful formation of the TG nanosheets. After covalent immobilization of MMC into TG, the thickness of nanocomposites increased to 4.3 nm (as shown in Figure S1). The cytotoxicity of the as-prepared TG against normal ocular APRE-19 cells was measured using the CCK-8 assay. Figure 1d shows that more than 95% of the cells survived when incubated in (up to) 50 µg/mL TG with ARPE-19 cells for 72 h. Even when 100 µg/mL TG was applied for 72 h, the cell survival rate remained higher than 80%. This result supports the observed high biocompatibility of TG.

The successful synthesis of TG was also confirmed by Raman spectra and is given in Figure S2. Three characteristic peaks associated with the presence of graphene nanosheets were observed in the spectrum of TG (red curve, Figure S2). These peaks were observed at 1320 cm^−1^ (D band, sp^3^ carbon), 1580 cm^−1^ (G band, sp^2^ carbon), and 2700 cm^−1^ (2D band). Compared to the spectrum of pristine graphite (blue curve, Figure S2), the relative intensity ratio of the D to G bands was seen to be much higher. This suggests that the interaction of TAT with graphene introduced more defects. Furthermore, a characteristic peak of TAT was observed at 940 cm^−1^ in the spectrum of TAT (black curve, Figure S2). This peak was visible in the spectrum of TG, and this result confirmed the successful synthesis of TG.

Ultraviolet–visible (UV-vis) spectroscopy was used to confirm the immobilization of MMC into TG. As seen in Figure S3, a prominent absorption peak at 260 nm was observed in the spectrum of TG (blue curve). Besides this band, an additional peak was seen at 358 nm in the spectrum of MMC-TG (red curve, Figure S3). This additional peak can be attributed to the characteristic absorption peak of MMC, which occurs at 363 nm in the spectrum of the pristine MMC (black curve, Figure S3). The blue shift of the MMC characteristic peak in MMC-TG indicated the interaction of MMC with TG.

The drug-loading and encapsulation efficiencies of the MMC-TG were found to be 22% and 55%, respectively, and were calculated using a standard curve of the composite’s UV absorption intensity measured at 363 nm for different MMC concentrations (Figure S4). The in vitro release of MMC from MMC-TG was tested in phosphate buffered saline (PBS) using a dialysis method and an incubator set to 37 °C. To perform this test, MMC-TG was placed in a dialysis bag containing various PBS solutions (set to pH 7.4 and 5.5). The content of MMC in the solution was measured by UV-vis for various periods of time. The MMC release rate was calculated by comparing the MMC standard release curve. Figure S5 shows a typical curve of various concentrations of MMC released from MMC-TG in solutions set at different pH values after 96 h of incubation. Forty-five percent of the MMC was released when the solution had a pH of 5.5, while 40% of the MMC was released at pH 7.4. The MMC release rate in the acidic solution was observed to be faster than that seen when a neural solution was tested. These results suggested an additional advantage for the antitumor effects of MMC-TG since the tumor microenvironment is usually acidic.

Further evidence confirming the successful synthesis of MMC-TG was obtained from the Fourier transform infrared (FTIR) spectra (Figure S6). The FTIR spectrum of the MMC-TG (blue curve, Figure S6) exhibited four characteristic peaks of the amido bonds at 3430 cm^−1^ (N–H stretching vibration), 1640 cm^−1^ (C=O stretching vibration), 1580 cm^−1^ (N–H bending vibration), and 1370 cm^−1^ (C–N stretching vibration). This supports the successful covalent linkage of MMC to TG. Characteristic peaks of MMC were seen in the spectrum of MMC-TG at 2960 cm^−1^ (C–H stretching vibration of aromatic rings) and in the range of 1500 to 1600 cm^−1^ (C=C stretching vibration of aromatic rings). This suggests the presence of MMC in the resulting MMC-TG.

**Figure 2:**
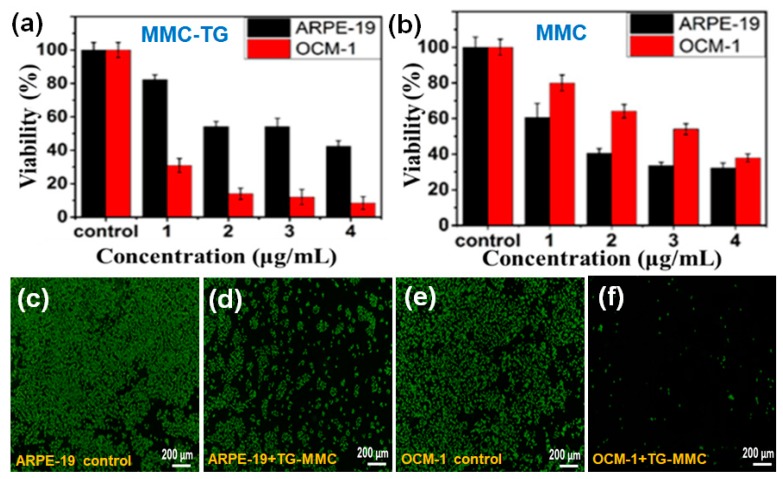
The cell viability of ARPE-19 and OCM-1 cells after being treated with (**a**) mitomycin C (MMC)-TG and (**b**) MMC over 72 h and fluorescence micrographs of Calcein-AM stained cells: (**c**) ARPE-19 cells, (**d**) ARPE-19 cells incubated with MMC-TG over 72 h, (**e**) OCM-1 cells, and (**f**) OCM-1 cells incubated with MMC-TG over 72 h. The concentrations in Figure 2a represent the concentrations of MMC in MMC-TG nanodrugs.

To test the targeted antitumor capability of the as-synthesized MMC-TG, a Transwell two-cell co-culture system was used [22]. Typically, normal ocular cells (such as ARPE-19 cells) and tumorous cells (e.g., OCM-1 cells) are co-cultured in the upper and lower compartments of the Transwell system, respectively. These two types of cells were co-cultured in the same culture media. The cell viability after the treatment of antitumor drugs was determined using a cell counting kit-8 (CCK-8) assay [23]. For the purpose of comparison, the as-prepared MMC-TG and pristine MMC were tested as the antitumor drug carriers. Figure 2a shows that MMC-TG is very toxic to the tumorous OCM-1 cells. Cytotoxicity of MMC-TG against OCM-l cells was found to be time and concentration dependent (Figure S7). An introduction of a very small amount of MMC-TG (even when the MMC-TG contained 1 µg/mL of MMC) with OCM-1 cells over 72 h resulted in a sharp decrease in the cell viability of tumorous OCM-l cells (less than 30%). In comparison, the survival rate of normal cells treated under identical conditions remained more than 80%. This result confirmed the highly targeted antitumor ability of the as-prepared MMC-TG nanodrugs, which showed strong toxicity to tumor cells but good biocompatibility with normal cells.

The cell viability of OCM-1 was further reduced to 5% when the cells were incubated with MMC-TG and consisted of 4 µg/mL MMC for 72 h, suggesting the strong tumor lethality of the MMC-TG nanodrugs. On the other hand, it was found that the pristine MMC induced indistinctive toxicity in both cell types (Figure 2b). Lower cell survival rates were obtained with normal human retinal pigment epithelial (ARPE-19) cells compared to tumor cells. Furthermore, more than 35% of tumor cells survived even when 4 µg/mL MMC applied over 72 h. This was much higher than that seen with the tumor cells treated with MMC-TG containing 4 µg/mL MMC over 72 h. This result supports the high targeted anticancer capability of MMC-TG and was attributed to the presence of TG, which consisted of an efficient tumor-targeting driver—TAT. To better identify the cancer suppression effect of MMC-TG, the IC50 of MMC-TG on OCM-1 cells was measured and compared to pure MMC that was tested as a negative control. The IC50 was calculated from data that is shown in Figure S8. In this figure, the OCM-1 cell viability vs. the various concentrations of MMC-TG and MMC can be seen. The IC50 of MMC-TG was observed to be 3.35 μg/mL, while that of thewas 12.27 μg/mL. The superior cancer suppression effect of the MMC-TG nanodrugs was thus confirmed.

Based on these cell viability results, samples containing 4 µg/mL MMC were tested with a calcein acetoxymethyl ester (Calcein-AM) staining assay. In this assay, the live cells appeared green after cells were stained by the fluorescent dye Calcein-AM. As shown in Figure 2c–f and Figure S9, when different types of cells were co-cultured with MMC-TG, the content of green fluorescence in the OCM-1 cells was significantly decreased with increasing culture time, while the fluorescence in the ARPE-19 cells remained unchanged. Less than 5% of live OCM-1 cells were seen in the group after incubation with MMC-TG over 72 h (Figure 2f). This is significantly lower than that seen with ARPE-19 cells (Figure 2d), suggesting that the cell survival rate of tumorous OCM-1 cells was much lower than that of normal ARPE-19 cells in the cell culture media containing MMC-TG. These results were consistent with the CCK-8 results shown in Figure 2a. For comparison, the fluorescence images of different Calcein-AM-stained cells co-cultured with MMC are shown in Figure S10. Both the ARPE-19 group and the OCM-1 group displayed gradually decreasing fluorescence intensity with culture time compared with that seen in the control groups. However, there is no significant difference between the fluorescence in the APRE-19 group and that in the OCM-1 group even after both these cell types were incubated with MMC even though less live cell fluorescence was observed in ARPE-19 cells than in OCM-l cells when these cells were co-cultured with MMC over 72 h. This indicates that the pristine MMC did not show any capability for tumor-targeting suppression. However, when TG was incorporated with MMC, a superior capability of tumor-targeting suppression was realized.

**Figure 3:**
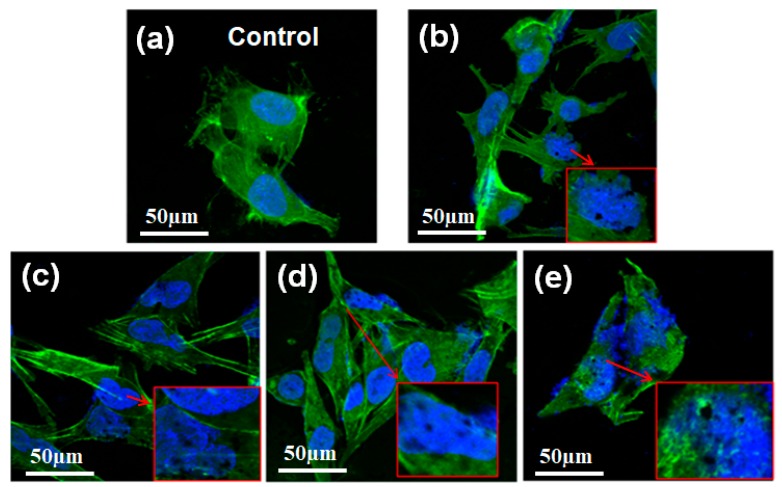
Confocal laser scanning microscopy images of OCM-1 cells marked by fluorescein isothiocyanate (FITC)-phalloidin and 4′,6-diamidino-2-phenylindole (DAPI) after incubation with TG over various time periods: (**a**) The control, (**b**) 6 h, (**c**) 24 h, (**d**) 48 h, and (**e**) 72 h. The insets show the magnified images of the corresponding cell parts.

To better understand the distribution of TG inside OCM-1 cells, we imaged the OCM-1 cells using a confocal laser scanning microscope (CLSM) labeled with fluorescein isothiocyanate (FITC)-phalloidin and 4′,6-diamidino-2-phenylindole (DAPI). As shown in Figure 3, the cytoplasm was stained by FITC-phalloidin and displayed a green color, while the nucleus was labeled with DAPI and showed a blue color. The presence of TG was visible inside the cells and even inside the nuclei after OCM-1 cells were incubated with TG over 6 h (Figure 3b). The amount of TG inside the nuclei significantly increased with an increase in co-culture time. The presence of TG inside the nuclei was still seen after 72 h incubation (Figure 3e). These results support the nuclear-targeting capability of the as-synthesized TG. To better identify the distribution of TG inside the nuclei of OCM-1 cells, the TG was also labeled and tested with an amine-reactive fluorescein derivative FITC (green fluorescence) via an amidation reaction between the carboxyl groups in the plasma-modified TG and the amino groups of FITC. The nucleus was further marked with a blue fluorescent DAPI, and the cytoplasm was stained by Texas Red^TM^-X phalloidin (red color, Invitrogen, Waltham, MA, USA). As shown in Figure S11, the presence of green fluorescent TG was visible in the nuclei of cells. This also supports the nuclear-targeted capability of the TG.

**Figure 4:**
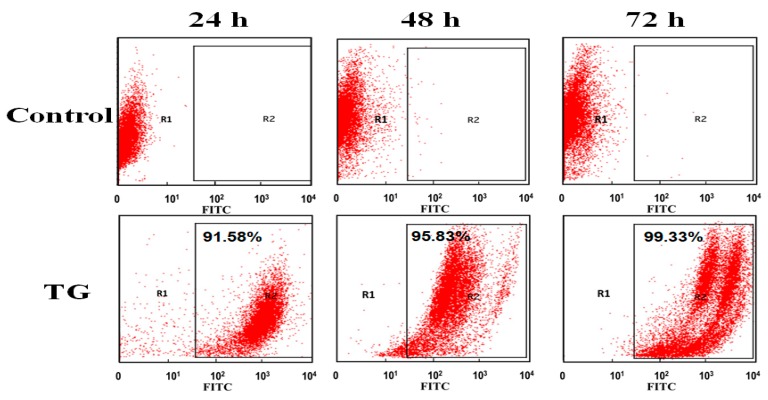
Flow cytometry analysis of OCM-1 cells incubated with FITC-stained TG for various time periods.

Flow cytometry was further also applied to the quantitative analysis of TG distribution inside OCM-1 cells. TG stained with FITC was incubated with OCM-1 cells for 24, 48, and 72 h. The as-prepared cells were collected and measured with flow cytometry. As shown in Figure 4, it was found that more than 91% of the cells were fluorescent after 24 h. This suggests that dominant cells contained TG after their co-incubation for 24 h. The content of fluorescent cells increased to 95% after 48 h and achieved almost 100% after 72 h. The results from flow cytometry further supported the highly targeted capability of TG to OCM-1 cells of TG and confirmed the high cell-membrane-penetrating capability of TG.

**Figure 5:**
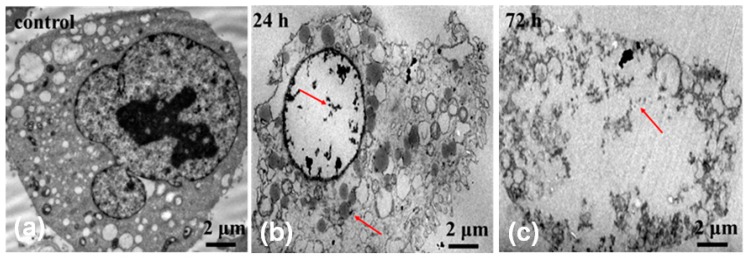
TEM micrographs of OCM-1 cells incubated with (**a**) the control, (**b**) 4 µg/mL MMC-TG for 24 h, and (**c**) 4 µg/mL MMC-TG for 72 h: The arrows in Figure 4b,c indicate the presence of MMC-TG inside the cells.

In addition, we carried out TEM characterization on OCM-l cells cultured with MMC-TG to further confirm the nuclear-targeting capability of the nanodrugs. Figure 5 shows TEM micrographs of OCM-1 cells incubated with 4 µg/mL MMC-TG over 24 and 72 h. When MMC-TG was co-cultured with OCM-1 cells after 24 h, the substance inside the nuclei dissolved significantly, and the morphology of the organelle became out of shape (Figure 5b). This suggests that the strong antitumor capability of MMC-TG and the damage of MMC-TG on tumor cells started from the cell nuclei. On the other hand, the residual MMC-TG nanomaterials were found both inside the nuclei and inside the cytoplasm as can be seen in Figure 5b. When the co-culture time was increased to 72 h, the karyotheca completely disappeared and the cytoplasm was also seen in the process of dissolution (Figure 5c). This confirmed the strong tumor lethality of the resulting MMC-TG. Overall, TEM results proved the presence of MMC-TG inside the tumor cell nuclei and further demonstrated the process of tumor apoptosis triggered by MMC-TG that started from its damage to the cell nuclei. This supported the excellent nuclear-targeting and antitumor capabilities of the as-prepared MMC-TG.

## 3. Discussion

The eye is an essential organ of a human body. More than 80% of our external information is obtained via our eyes [24]. Hence, visual health is associated with a person’s quality of life [25] and ocular malignant tumors such as chorodial melanoma (CM) significantly damage visual health and quality of life, as well as threaten the patients’ life. The greatest challenge for clinical treatment of this disease is its ease of metastasis. CM has a five-year survival rate that is less than 50% [26]. Though many advanced techniques, including some that use nanotechnology, have been developed to improve the controlled release of antitumor drugs and its specific suppression effects on CM [27,28], the risk of tumor cell metastasis remains high.

In this work, TAT, a type of cationic cell-penetrating peptide, was used to better target the nuclei of cancer cells. In particular, its ability to penetrate the cell membrane and its nuclear membrane was tested. In order to optimize the targeting and drug-loading impact, two-dimensional graphene nanosheets were bound with TAT via an EFBM procedure developed by the author’s research group. This method provided an environmentally friendly and efficient procedure to prepare TAT-functionalized graphene (TG) that had minimal structural defects. No chemical redox reaction was involved during its fabrication. A common antitumor drug, MMC, was then introduced into the TG nanosized drug-carrier. To the best of our knowledge, MMC has not been reported elsewhere before for the treatment of uveal melanoma including CM. This may be due to the myelosuppression issues related to MMC administration and to the inconvenience of administrating fundus [29]. Some studies have reported the effective treatment of conjunctival melanoma using MMC [30], but the side effects of this treatment on normal cornea, conjunctiva, sclera, ciliary body, lens, and trabecular net tissues are unavoidable [31]. Another report elsewhere demonstrated that the impact of arterial embolization therapy using MMC on the liver metastasis of melanoma prolonged patient survival [32]. This is encouraging and lead us to believe that the resistance of uveal melanoma against MMC can be resolved by improving the nuclear-targeting capability of MMC for melanoma cells.

The resulting MMC-TG nanodrug had more than a 95% specific tumor suppression rate and, at the same time, a low toxicity towards normal cells. This was confirmed by both a Transwell two-cell co-culture system and Calcein-AM live cell-staining tests. Its high impact on tumor inhibition was attributed to the outstanding targeted nuclear penetration capability of TG. CLSM and TEM results confirmed the presence of TG-based nanomaterials inside cancer cell nuclei. This suggests that the TG nanocarrier was able to carry the MMC drug into the nuclei of tumor cells. The TEM results indicate that the apoptosis of tumor cells was caused by the lysis of substances within the cell’s nuclei and confirm that most MMC-TGs were accumulated inside the nuclei first. Thus, the damage of nuclear substances induced by the nanodrug occurs at this initial stage. Since the cell nucleus is the source of gene inheritance and transcription, it has not escaped our notice that preventing tumor cells from initiating metastasis, as seen in this proposed treatment, is a very useful property. The as-synthesized novel nuclear-targeting drug delivery system reported here promises the great possibility of resolving the tumorous metastasis challenge that has troubled both fundamental researchers and clinical doctors treating this usually fatal disease. The myelosuppression issue of MMC and the biosafety problem of graphene in other human body systems have yet to be settled. Thus, the best administration routine for clinical utilization of MMC-TG, in our opinion, is via topical administration.

## 4. Materials and Methods

### 4.1. Reagents and Instruments

Graphite flakes were kindly provided by Haida Corporation (Qingdao, China). TAT was purchased from GL Biochem Ltd. (Shanghai, China). 1-ethyl-3-(3-dimethylaminopropyl) carbodiimide hydrochloride (EDC• HCl), N-hydroxylsuccinimide (NHS), and Mitomycin C (MMC) were obtained from Sigma-Aldrich (St. Louis, CA, USA). Transwell plates were supplied by Corning Incorporated. The CCK-8 was sourced from Dojindo Company (Kumamoto, Japan). OCM-1 and ARPE-19 cells were purchased from the American Type Culture Collection (ATCC) (Manassas, VA, USA). DMEM/F12 culture medium, 1640 culture medium, Fetal Bovine Serum (FBS), and trypsin were obtained from Gibco (Waltham, MA, USA).

The physiochemical properties of nanomaterials were characterized using Raman spectroscopy (ALMEGA XR, Thermo Scientific, Waltham, MA, USA), FTIR (Nicolet 6700, Thermo Scientific, Waltham, MA, USA), and UV-vis (Cary 100, Agilent Technologies, Chandler, AZ, USA). The morphologies of nanomaterials were measured using an AFM (Multimode 8, Bruker, Billerica, MA, USA) in a tapping mode.

### 4.2. Preparation of TG Nanocarriers and MMC-TG Nanodrugs

TG was synthesized via a self-discovered, one-step EFBM method at room temperature. Typically, 10 mg of TAT and 10 mg of graphite flakes were added to a 400-mL milling capsule. These mixes were vigorously shaken at the speed of 400 rpm for 4 h in a planetary ball-milling machine (Nanda Instrument Plant, Nanjing, China). Twenty mL of deionized (DI) water was then used to remove the milled mixture from the capsule. The samples were subsequently centrifuged at 1000 rpm for 10 min to remove unreacted graphite in the sediment. The supernatant was further treated by centrifuging at 8000 rpm for 10 min. This step was repeated 3 more times to remove any unreacted TAT. The thus treated TG was dispersed in deionized water for further testing. To prepare the TG powder, the as-synthesized TG dispersion was freeze-dried for 24 h. The as-prepared nanomaterial powder was treated with plasma in acetic acid vapor at 50 W for 5 min to introduce carboxyl groups to the TG surface so that the MMC could be immobilized. TG was then soaked in the aqueous solution containing NHS and EDC for 2 h to activate its carboxyl groups. MMC was then covalently loaded to TG via an amidation reaction run at 4 °C for 48 h. The formed MMC-TG was gathered by centrifuging at 8000 rpm for 10 min. This step removed residual NHS, EDC, and unreacted MMC.

### 4.3. The Transwell Two Cell Co-Culture System

The Transwell system consisted of a microporous membrane between two compartments. This was used to co-culture two kinds of cells in the same culture medium simultaneously. In this work, normal ARPE-19 cells were seeded in the upper chamber of the Transwell plate and tumorous OCM-1 cells were cultured in the lower compartment. MMC and MMC-TG dispersed in culture medium were added to the Transwell system at MMC densities of 1 µg/mL, 2 µg/mL, 3 µg/mL, and 4 µg/mL after the cells were attached to each compartment. The cell viability was evaluated using the cell counting kit-8 (CCK-8) for different incubation periods. The live staining of cells were performed using a calcein acetoxymethyl ester (Calcein-AM) stain kit, followed by its fluorescence microscopy characterization.

### 4.4. Confocal Laser Scanning Microscopy

TG was incubated with OCM-1 for 6 to 72 h. Cells were fixed in 4% paraformaldehyde for 15 min, followed by permeabilizing with 0.1% Tritonx-100 for 5 min. Cell cytoplasm was stained with fluorescein isothiocyanate (FITC) labeled with phalloidin. The cell nucleus was stained with 4,6-diamidino-2-phenylindole (DAPI) for 5 min. Distribution of nanomaterials inside cells was identified using a confocal laser scanning microscope (LSM710, Zeiss, Heidenheim, Germany).

### 4.5. Flow Cytometry

Flow cytometry measured the percentage of OCM-1 cells containing TG. Measurement was based on the fluorescence intensity of the cells. OCM-1 cells were seeded in 6-well culture plates at a cell density of 20 × 10^3^ cells/well. The plates were placed in a cell culture incubator for 24 h to allow the cell to spread. Four µg/mL of TG stained with FITC was then co-cultured with OCM-1 cells for various time periods. The cells were harvested at 24, 48, and 72 h. Cells were washed three times with PBS before measurement with the flow cytometry.

### 4.6. Transmission Electron Microscopy

OCM-1 cells were pre-cultured with 4 µg/mL MMC-TG for 24 and 72 h. Cells were collected and prefixed with 2.5% glutaraldehyde at 4 °C for 15 min. Cells were then postfixed with 1% osmic acid at 37 °C for 1 h, followed by staining with 1% uranyl acetate at 37 °C for 1 h. Cells were gradient dehydrated with different acetone solutions at various concentrations and then soaked in embedding liquid at 37 °C overnight. The cells were then embedded in a polymerizing liquid to allow them to be solidified. The cell blocks were sliced to ultrathin sections for their characterization with TEM (H-7500, Hitachi, Japan).

## 5. Conclusions

In summary, we successfully prepared a highly efficient nuclear-targeting antitumor drug-carrier formed from MMC-immobilized TG. The strong nuclear-targeting capability was achieved by the incorporation of TAT with two-dimensional nanosheet graphene using an environmentally friendly edge-functionalized ball-milling method. MMC was covalently immobilized onto TG. The as-synthesized MMC-TG had a strong suppression capability (with a more than 95% suppression efficacy) towards tumorous OCM-1 cells and a low toxicity against normal ARPE-19 cells (when two types of cells were co-cultured in the same culture media containing MMC-TG using a Transwell two-cell co-culture system). The nuclear targeting capabilities of both TG and MMC-TG were confirmed by CLSM and TEM measurements. TEM images further demonstrated the dynamic apoptosis of tumor cells induced by MMC-TG, which was observed to start from the lysis of cell nuclei. These results suggest that the as-synthesized MMC-TG is a good candidate as an antitumor drug carrier system, as it exhibited good tumorous nuclear targeting capability and a strong tumor suppression efficacy. Thus, this work suggests a new approach to the development of novel antitumor drugs.

## Supplementary Materials

Supplementary materials can be found at https://www.mdpi.com/1422-0067/20/18/4454/s1.

## Abbreviations

TAT	Transactivating transcriptional activator
TG	TAT peptide-functionalized graphene
MMC	Mitomycin C
OCM	Ocular choroidal melanoma
RPE	Retinal pigment epithelial
CM	Choroidal melanoma
CPP	Cell-penetrating peptide
EFBM	Edge-functionalized ball-milling
MMC-TG	MMC-loaded TG
EDC	1-ethyl-3-(3-dimethylaminopropyl) carbodiimide
NHS	N-hydroxylsuccinimide
CCK-8	Cell counting kit-8
FBS	Fetal bovine serum
AFM	Atomic force microscopy
TEM	Transmission electron microscopy
CLSM	Confocal laser scanning microscopy
FTIR	Fourier transform infrared spectroscopy
UV-vis	Ultraviolet–visible spectroscopy
Calcein-AM	Calcein acetoxymethyl ester
PI	Propodium iodide
DAPI	4’,6-diamidino-2-phenylindole
