# Supplementary material for: The Use of TAT Peptide-Functionalized Graphene as a Highly Nuclear-Targeting Carrier System for Suppression of Choroidal Melanoma
Source: Int J Mol Sci. 2019 Sep 10;20(18):4454. doi: 10.3390/ijms20184454 (PMC6769650; doi:10.3390/ijms20184454)
Supplement: Supplementary file 1 [file ijms-20-04454-s001.pdf]

SUPPORTING INFORMATION

The use of TAT peptide functionalized graphene as a highly nuclear-targeting carrier system for suppression of choroidal melanoma

Suyan Shan[1], Shujuan Jia[1], Tom Lawson[2], Lu Yan[1], Mimi Lin [1,*], and Yong Liu[1,*]

[1]*Labaratory of Nanoscale Biosensing and Bioimaging, School of Ophthalmology and Optometry, School of Biomedical Engineering, Wenzhou Medical University, 270 Xueyuanxi Road, Wenzhou, Zhejiang 325027, China*

[2]*ARC Centre of Excellence for Nanoscale Biophotonics, Macquarie University, Sydney, NSW 2109, Australia*

*Emails: linmimi815@wmu.edu.cn, yongliu@wmu.edu.cn

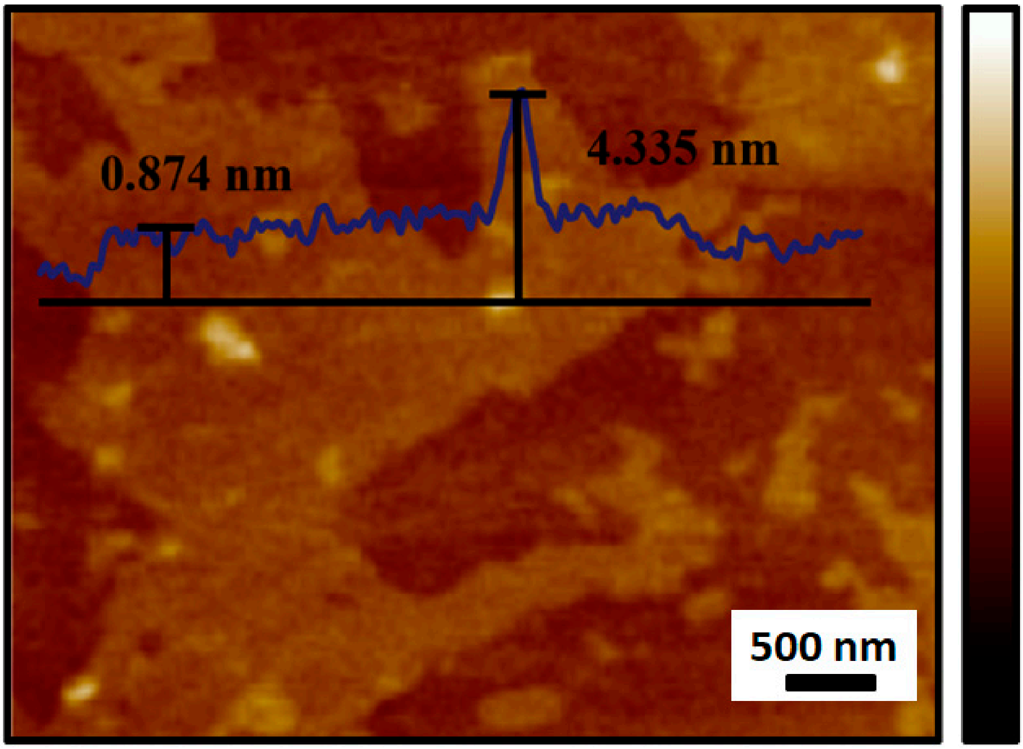

Figure S1. AFM micrograph of the as-prepared MMC-TG.

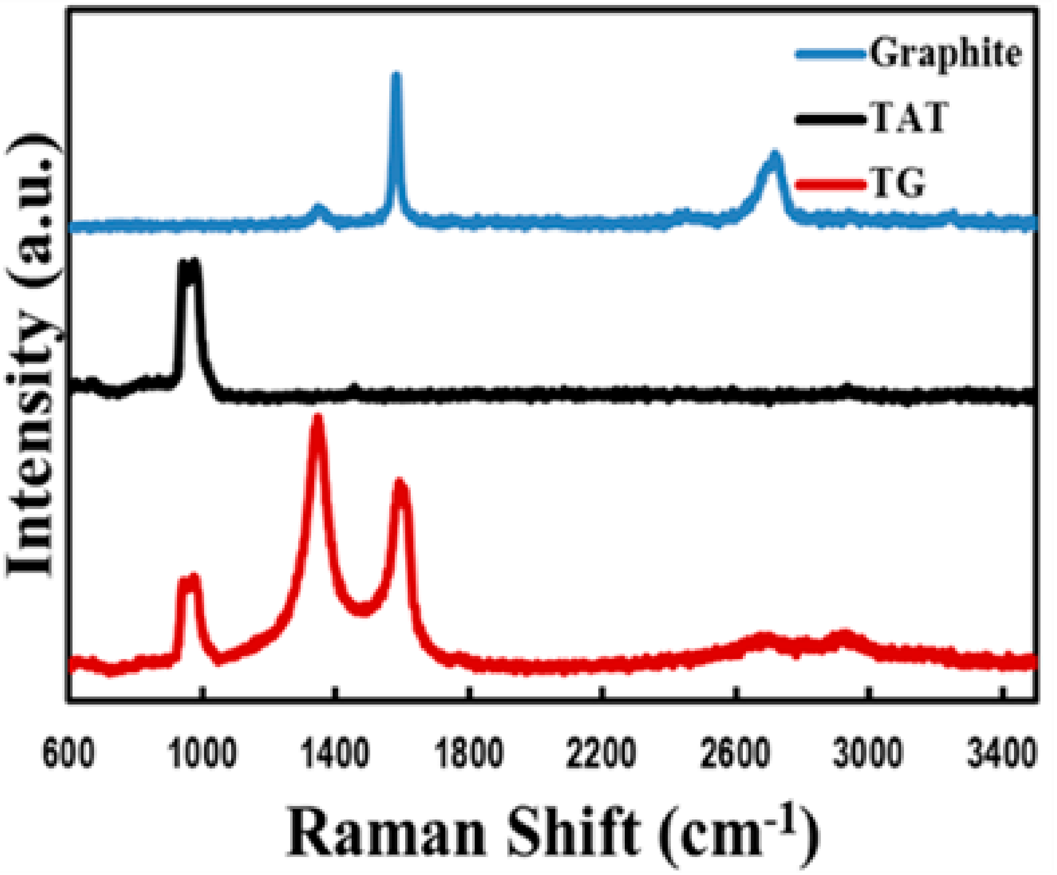

Figure S2. Raman spectra of (a) the pristine graphite, (b) the pristine TAT, and (c) the obtained TG.

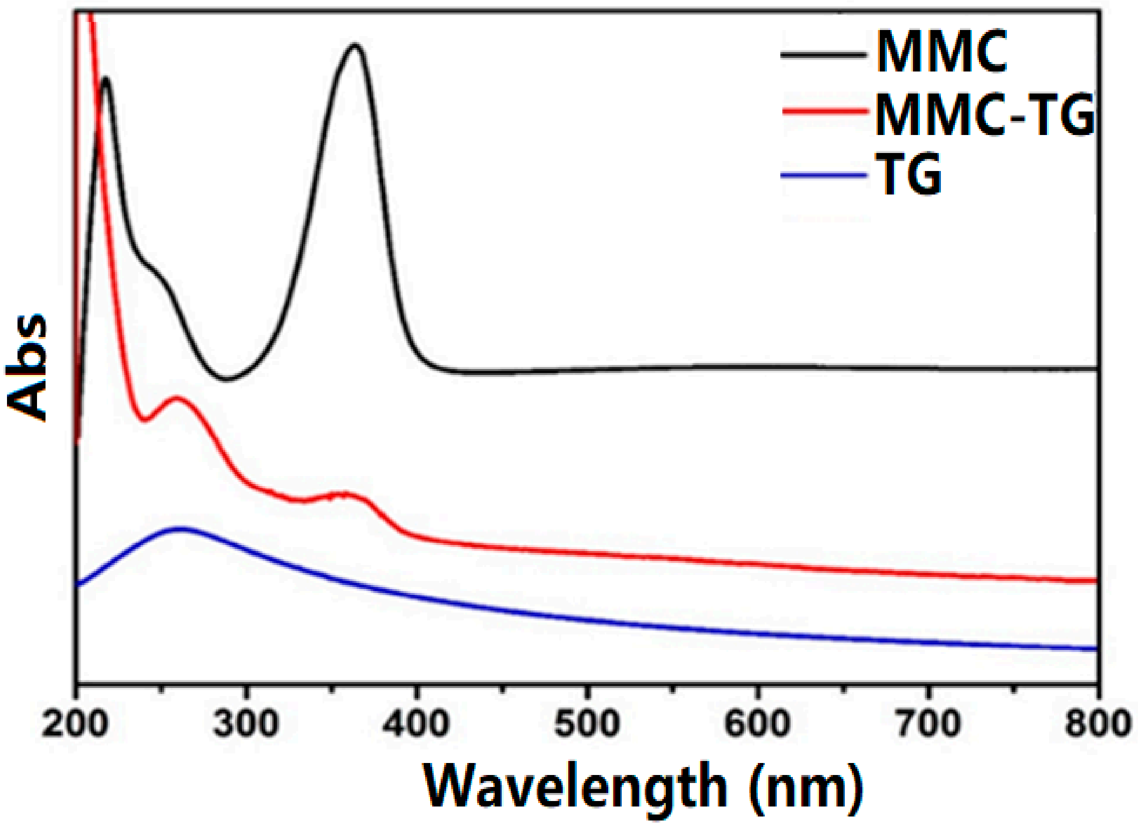

Figure S3. UV-vis spectra of (a) the pristine MMC, (b) the as-synthesized MMC-TG, and (c) TG.

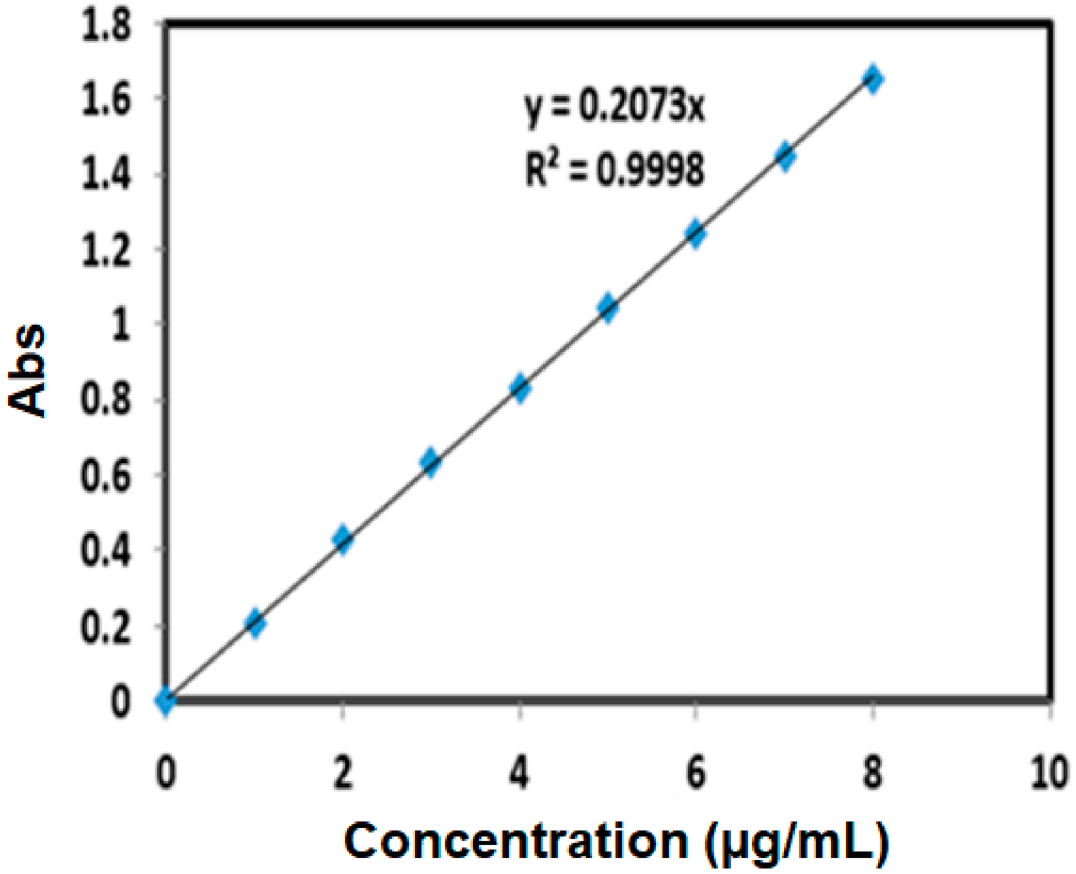

Figure S4. The standard curve of the UV absorption intensity at 363 nm *vs* the MMC concentration.

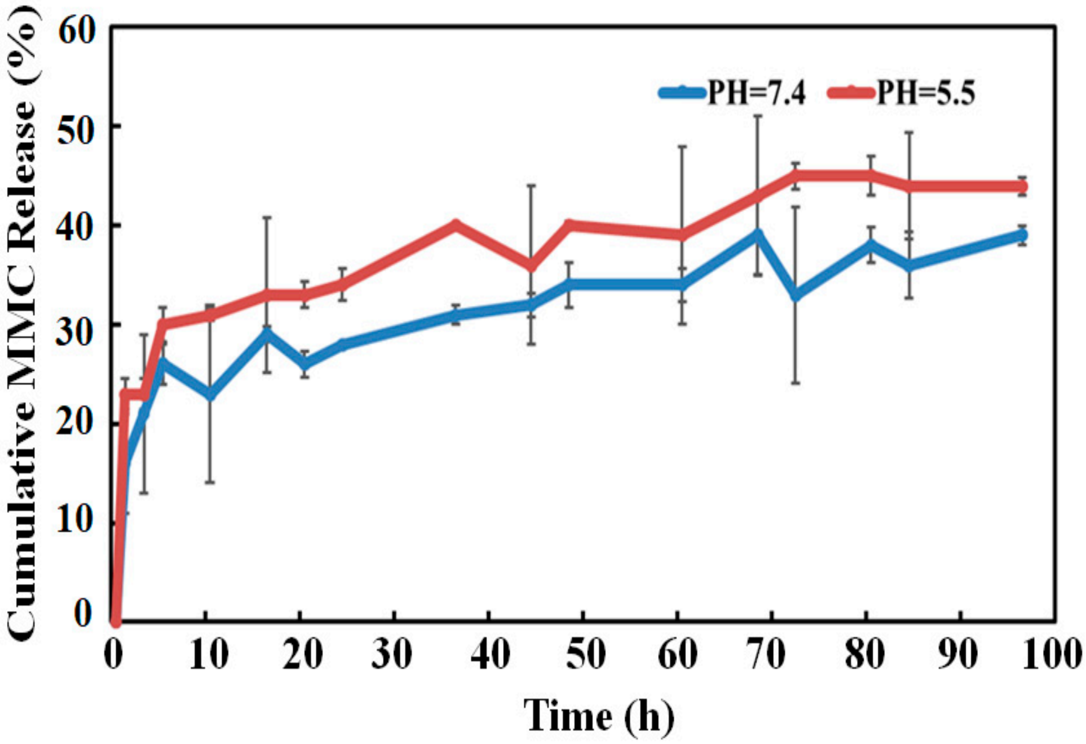

Figure S5. The typical release of MMC from the MMC-TG nanodrug in PBS solutions set at different pH values.

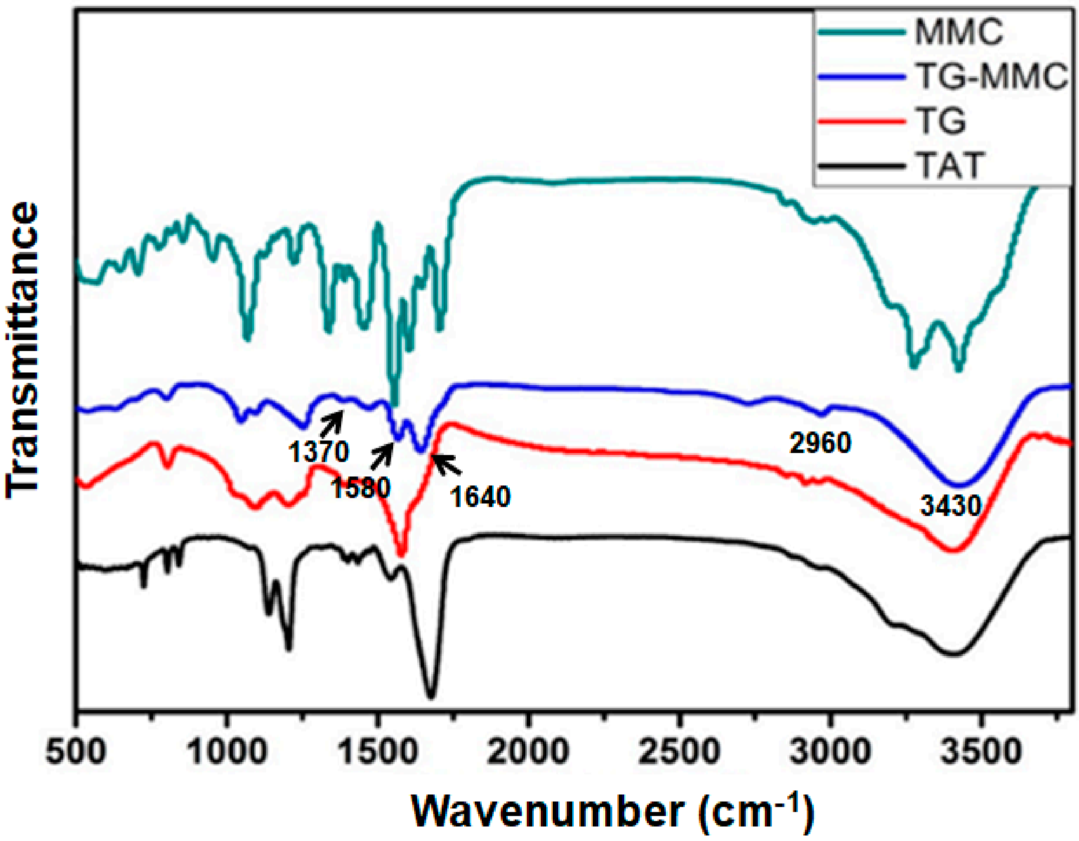

Figure S6. FTIR spectra of (a) the pristine MMC, (b) the prepared MMC-TG, (c) the synthesized TG, and (d) the pristine TAT.

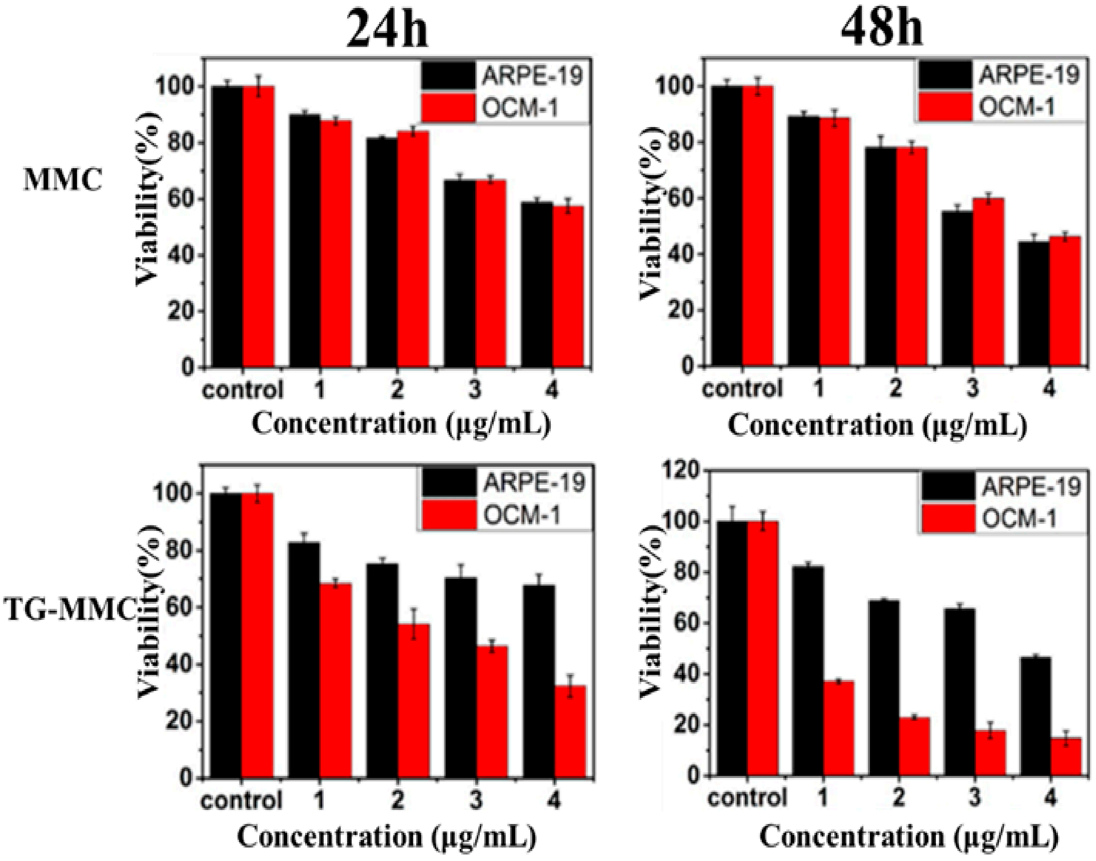

Figure S7. The cell viability of ARPE-19 and OCM-1 cells after being treated with MMC and MMC-TG over 24 and 48 hrs, respectively.

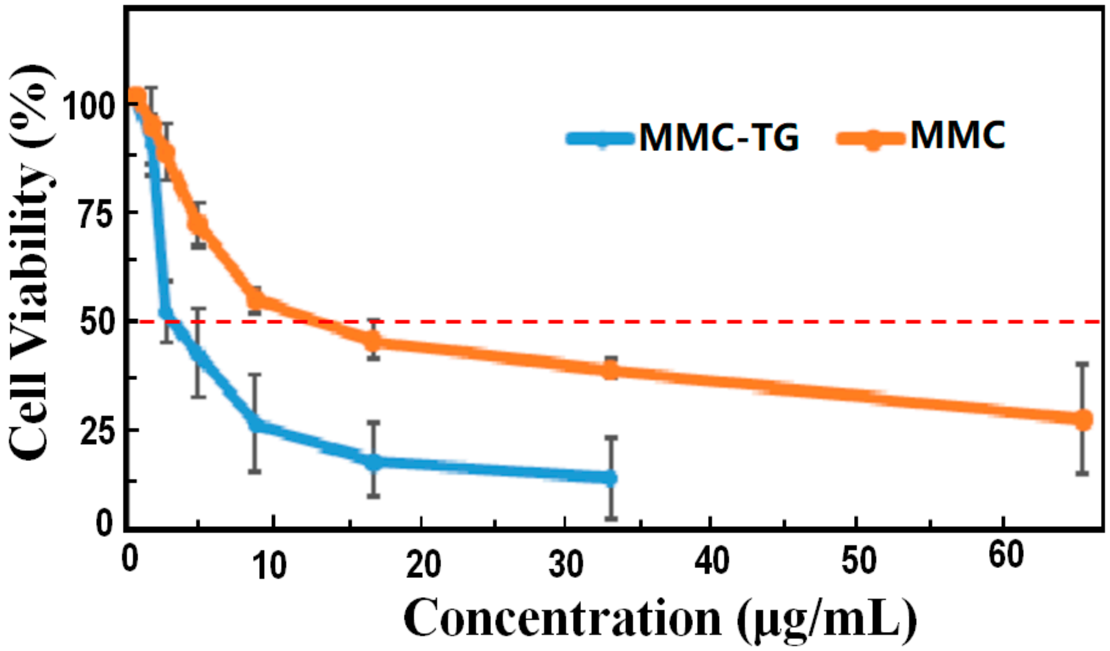

Figure S8. Cell viability of OCM-1 cells after being treated with the MMC-TG nanodrug compared to the pristine MMC at various concentrations.

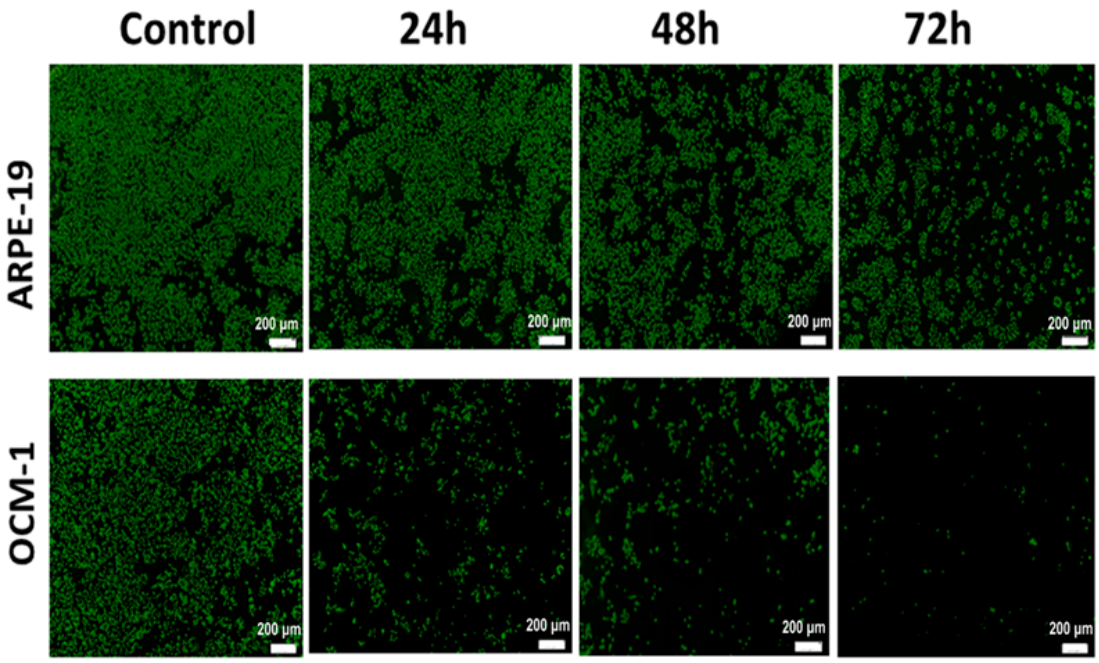

Figure S9. Fluorescence micrographs of various cells stained by Calcein-AM dyes and incubated with MMC-TG over 24, 48 and 72 hrs.

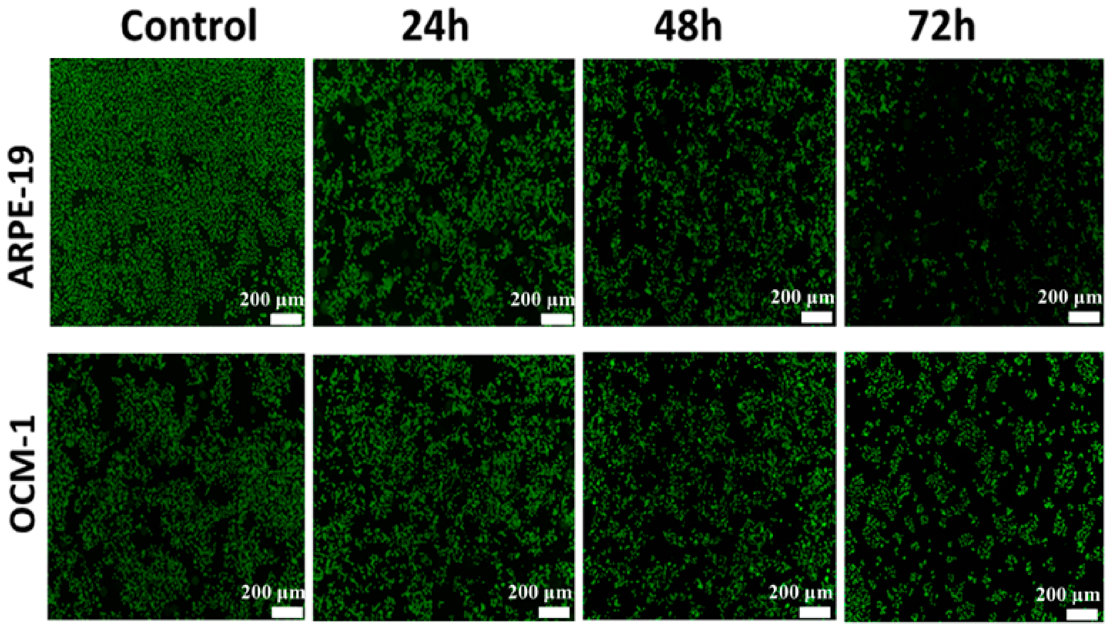

Figure S10. Fluorescence micrographs of different cells stained by Calceim-AM dyes and incubated with MMC over 24, 48, and 72 hrs.

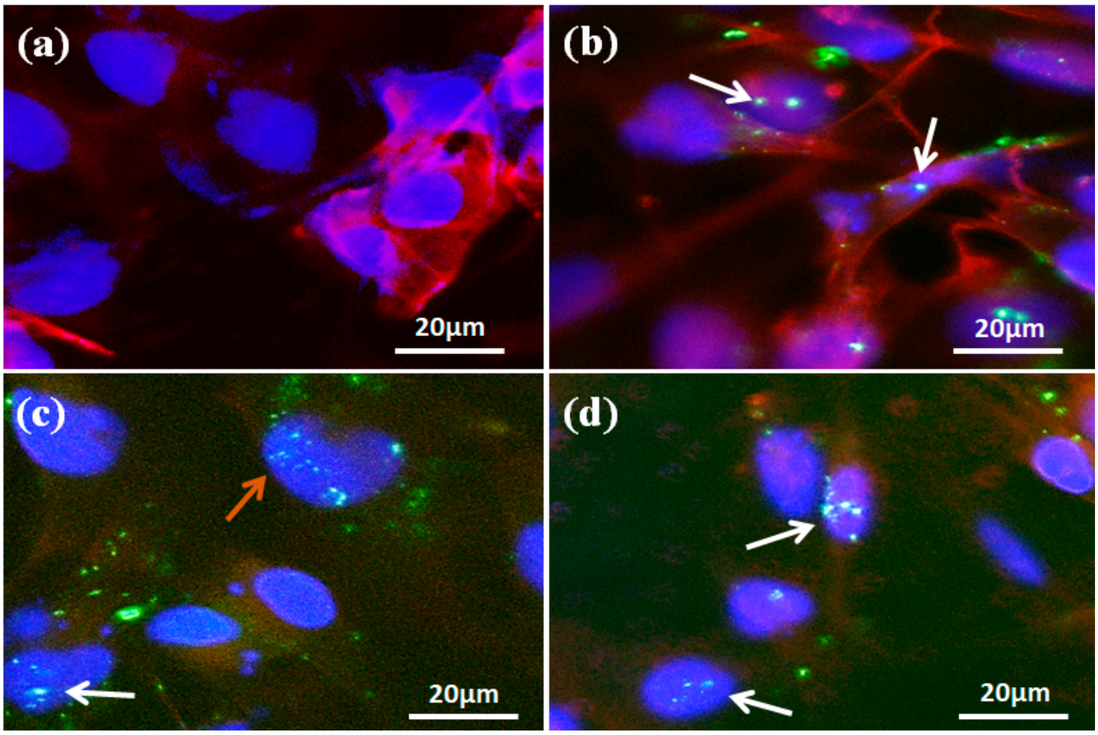

Figure S11. Confocal laser scanning microscopy images of OCM-1 cells marked by Taxas Red TM-X phalloidin (red color, cytoplasm) and DAPI (blue color, nucleus) after incubation with TG stained with FITC (green color) over various periods. (a) The control, (b) 24 hrs, (c) 48 hrs, and (d) 72 hrs. Arrows indicate the presence of TG inside the cell nuclei.
